# Hepatitis E Virus RNA Presence in Wild Boar Carcasses at Slaughterhouses in Italy

**DOI:** 10.3390/ani11061624

**Published:** 2021-05-31

**Authors:** Mario Forzan, Maria Irene Pacini, Marcello Periccioli, Maurizio Mazzei

**Affiliations:** 1 Department of Veterinary Sciences, University of Pisa, Viale delle Piagge 2, 56124 Pisa, Italy; mario.forzan@unipi.it (M.F.); mariairene.pacini@phd.unipi.it (M.I.P.); 2 Azienda USL Toscana Sud Est, Dipartimento di Prevenzione, Unità Funzionale di Sanità Pubblica Veterinaria e Sicurezza Alimentare Zona Distretto Colline dell’Albegna, Orbetello, 58013 Grosseto, Italy; marcello.periccioli@uslsudest.toscana.it

**Keywords:** hepatitis E virus, wild boar, zoonosis, public health

## Abstract

**Simple Summary:**

Hepatitis E virus (HEV) is a worldwide diffused pathogen responsible for acute hepatitis of humans. Transmission of the pathogen is mostly related to the consumption of contaminated food and water. Although initially the disease was contained in developing countries, in recent years autochthonous infections have been reported in several industrialised countries. A different epidemiological pattern of transmission has been highlighted; while in Africa and Asia transmission is mainly due to waterborne outbreaks caused by low sanitation standards, in Europe and other industrialised countries, the disease has mainly spread due to consumption of raw or undercooked meat and seafood. Although HEV has been identified in several domestic and wild animal species, pigs and wild boar, appear to play a distinct role mainly acting as a reservoir of the pathogen. In this study, we monitored the presence of HEV in carcasses and livers of wild boar sampled in Tuscany at the slaughterhouse following hunting activities. Our data indicate the presence of the pathogen in the liver and the carcasses, suggesting cross-contamination. This evidence highlights the importance of maintaining safety control measures to avoid the spreading of HEV infection.

**Abstract:**

Hepatitis E virus (HEV) is a waterborne and foodborne pathogen largely spread around the world. HEV is responsible for acute hepatitis in humans and it is also diffused in domestic and wild animals. In particular, domestic pigs represent the main reservoir of the infection and particular attention should be paid to the consumption of raw and undercooked meat as a possible zoonotic vehicle of the pathogen. Several studies have reported the presence of HEV in wild boar circulating in European countries with similar prevalence rates. In this study, we evaluated the occurrence of HEV in wild boar hunted in specific areas of Tuscany. Sampling was performed by collecting liver samples and also by swabbing the carcasses at the slaughterhouses following hunting activities. Our data indicated that 8/67 (12%) of liver samples and 4/67 (6%) of swabs were positive for HEV RNA. The presence of HEV genome on swabs indicates the possible cross-contamination of carcass surfaces during slaughtering procedures. Altogether, our data indicated that it is essential to promote health education programmes for hunters and consumers to limit the diffusion of the pathogen to humans.

## 1. Introduction

Hepatitis E virus (HEV) is a widespread pathogen causing viral hepatitis and it is considered an emerging pathogen in industrialized countries [1]. HEV is a small, enveloped, single-stranded positive-sense RNA virus classified in the Orthohepevirus A genus in the *Hepeviridae* family [2]. Based on the whole-genome phylogenetic analysis, members of the species Orthohepevirus A are divided into eight genotypes (Gt) from HEV-1 to HEV-8. The first four Gts are the most common and include several subtypes, six subtypes (1a–1f) within HEV-1, two (2a and 2b) within HEV-2, 11 (3a–3j and 3ra) within HEV-3 and nine (4a–4i) within HEV-4 [2]. Genotypes 1 and 2 are restricted to humans and often associated with large outbreaks in developing countries with poor sanitary standards and are mainly transmitted by the faecal–oral route. Genotypes 3 and 4 are mostly endemic in both developing and industrialised countries where are responsible for infection in humans, pigs and other animal species. HEV-3 and 4 are considered zoonotic and have been responsible for sporadic cases of hepatitis E in humans [3,4,5,6]. The human infection is characterised by acute hepatitis that rarely leads to death, except in pregnant women, where the fatality rate is up to 25% [4]. In Europe, HEV-3 is the most common Gt in humans, with different seroprevalences ranging from 20–86% in various countries depending on age groups [7,8]. Due to the wide diffusion of HEV in wild and domestic species, multiple epidemiological transmission patterns are described from breeding farm to slaughterhouses, in processing meat/seafood industries and wastewater management, leading HEV to be considered as a global public health issue [9]. Furthermore, an association between occupational exposure to swine and human HEV seropositivity was reported for personnel working in the slaughterhouse, forestry workers, hunters or farmers [1,2,8,10,11,12]. At the slaughterhouse, HEV has been identified in pig derived samples, in particular on faeces, bile, liver and other internal organs that can be characterized by high viral loads, but also in blood and muscles [8,9,13,14,15,16,17]. Therefore, during slaughtering, particular attention should be paid to the processing of the carcasses and organs, which should be kept separated to avoid cross-contamination [14,15,17,18,19,20]. Moreover, dedicated equipment and utensils, particularly knives, should be used only for their specific operations [8]. Several studies indicate that wild boar could play an important epidemiological role as a reservoir of HEV and that the consumption of undercooked or raw wild boar meat could be responsible for HEV infection in humans [8,21,22,23,24,25,26,27]. Indeed, in developed countries, autochthonous HEV infections are largely due to contact with infected animals, in particular pig and wild boar, and to ingestion of contaminated raw or undercooked meat and seafood [6,8,28].

Epidemiological studies conducted in wild boar populations across Europe countries, including Italy, recorded molecular prevalences ranging from 0.3% to 68.2% and seroprevalences ranging from 12.5% to 57.4%, confirming the wide diffusion of HEV [6,7,22,23,27,29,30]. No specific microbiological criteria for game meat exist as yet in the EU legislation. Regulation (EC) no.2073/2005, which states values for total viable count (TVC) and Enterobacteriaceae in the carcasses of pigs and ruminants, can be used for game meat, and no other inspective criteria are required. Moreover, HEV-infected animals are asymptomatic and consequently it is not possible to identify infected animals by antemortem evaluation or inspection of carcasses. Therefore, although direct animal contact may or not play a principal role in HEV transmission [21,31], operations such as skinning and disembowelling performed by hunters on wild boar carcass could be an efficient transmission route if no protective gloves are worn, especially if abrasions on the skin of operator are present [32]. If slaughterhouses have a key role in HEV diffusion, the possibility of carcass contamination during wild boar slaughtering should be investigated as is done in the pig industry.

In this work, wild boar liver samples and swabs collected during slaughtering directly from carcasses were tested to evaluate the presence of HEV genomes by molecular assays. A correlation among tissue and swab positive samples was conducted to verify the possibility of contamination of carcass during slaughtering activities.

## 2. Materials and Methods

### 2.1. Samples Collection

**Figure 1:**
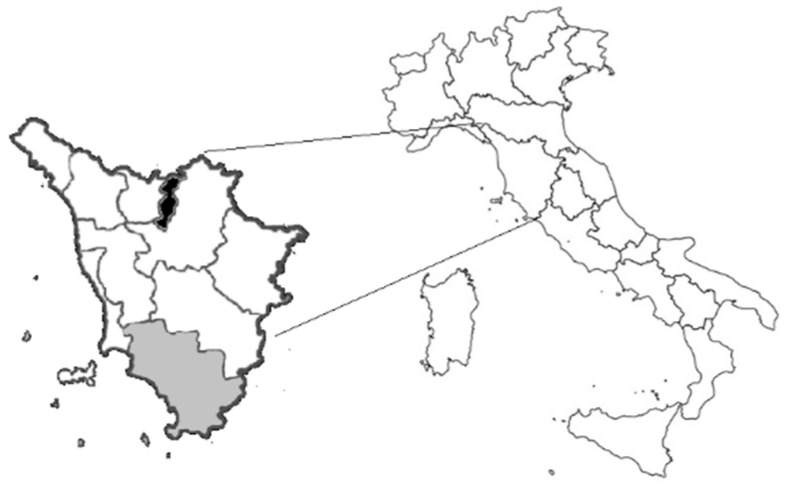
Map of sampling area: Detailed map of Tuscany; black and grey area represent the Prato and Grosseto provinces, respectively.

Samples were collected from November 2020 to January 2021 during wild boar hunting season, in the provinces of Grosseto and Prato (Tuscany, Italy) (Figure 1), two areas characterized by the abundant presence of wild boar and other wild ungulates. The hunting activity was carried out following the Regional Hunting Law (Regolamento di attuazione della legge regionale 12 gennaio 1994 n◦ 3 DPGR 48/R/2017).

After hunting, wild boar were transported to a game-meat cutting establishment for the slaughtering procedures. During those procedures, a little portion of the liver from each animal was collected. Moreover, after the evisceration procedures, the empty abdominal cavity and muscles of each carcass were swabbed using a wide-tipped polypropylene swab. No rupture of gastrointestinal tract was recorded for any individual. After collection, the swabs were placed in sterile tubes. The liver samples and the swabs were then transported to the Department of Veterinary Science (University of Pisa) and stored at −20 °C until molecular analysis.

A total of 67 wild boar were sampled in 11 working days during slaughtering activities. Age, sex, hunting area and date of sampling were recorded for each sampled animal. Age was evaluated through the degree of tooth eruption and the wear of teeth of the lower jaw [33].

### 2.2. RNA Extraction

On liver samples, the RNA extraction was performed using the RNeasy Mini Kit (Qiagen, Hilden, Germany), using 30 mg of tissue following manufacturer’s instructions. Swabs were thawed and soaked in 2 mL of sterile Phosphate Saline Buffer (PBS) in 15 mL tubes, incubated for 2 h at room temperature (RT), and finally centrifuged at 3000× *g* for 10 min. The RNA extraction was performed on 280 µL of the supernatant using QIAamp Viral Mini Kit (Qiagen, Hilden, Germany) following manufacturer’s instructions. After extraction, all RNAs were quantified using NanoDrop (Thermo Fisher Scientific, Waltham, MA, USA).

### 2.3. Generation of HEV Standard Curve

To generate an RNA standard curve for the RT-qPCR assay, a 296 bp synthetic oligo representing part of the ORF3 spanning the real-time amplicon was cloned into the pcDNA3.1(+) vector under the T7 polymerase promoter (GenScript Biotech, Leiden, The Netherlands). DNA plasmid was linearized by Hind III digestion (NEB, Ipswich, MA, USA), and purified by the Mini-elute reaction clean-up kit (Qiagen, Hilden, Germany). Linearised DNA was then used for in vitro transcription using MAXIscript SP6/T7 kit (Thermo Fisher Scientific, Waltham, MA, USA) following manufacturer instructions. Newly generated RNA was purified using the Minielute RNeasy clean-up kit (Qiagen, Hilden, Germany). RNA concentration was estimated by NanoDrop (Thermo Fisher Scientific, Waltham, MA, USA) and serially diluted to yield 6 dilution points from 4 × 10^6^ to 4 × 10^1^ RNA molecules/μL. Dilutions were employed to generate the standard curve used for the TaqMan real-time PCR assay.

### 2.4. RT-qPCR

RNAs extracted from liver and swabs were used as templates to perform an RT-qPCR using the Luna Universal Probe One-Step RT-qPCR Kit (NEB, Ipswich, MA, USA) targeting a conserved gene of HEV ORF3, using primers and probes previously described [34]. Each sample and each point of the standard curve were assayed in duplicate. The viral load was calculated as viral copies number/100 ng of RNA.

### 2.5. PCR

A nested RT-PCR targeting a 345 bp portion of the ORF2 gene [35] was attempted on all the samples for confirming the results of the real-time PCR and for sequencing. The first PCR was performed using One-step RT PCR kit (Qiagen, Hilden, Germany), the nested PCR using Hot Start Taq Plus Master Mix kit (Qiagen, Hilden, Germany).

### 2.6. Sequence and Phylogenetic Analyses

Nucleotide sequence analysis was applied to obtain phylogenetic information on the viral strains circulating in the studied areas and to verify the viral homology of sequences obtained from liver samples and the correlated swabs. For phylogenetic analysis, a set of the most representative GenBank available sequences were identified. Phylogenetic trees were generated using maximum-likelihood methods, as available in the MEGA6 software package [36]. The evolutionary history was inferred by using the Maximum Likelihood method based on the Tamura–Nei model. The bootstrap test was applied to calculate the percentage of replicate trees in which the associated taxa clustered together (100 replicates).

## 3. Results

A total of 67 wild boar were sampled and classified by sex as 26/67 females (39%) and 41/67 males (61%), and by age in 15/67 subadults (22%) (11–24 months) and 52/67 adults (>24 months) (78%). The quantitative and qualitative analysis performed on extracted RNAs showed that all liver samples and swabs were suitable for subsequently molecular analysis with an RNA concentration ranging from 53 to 1129 ng/ul for liver samples and from 104 to 136 ng/ul for swabs. The RT-qPCR identified 8/67 (12%) positive liver samples and 4/67 (6%) positive swabs. Positive samples belonged to 10 animals, 5/10 (50%) females and 5/10 (50%) males, 2/10 (20%) sub-adults and 8/10 (80%) adults (Table 1).

**Figure 2:**
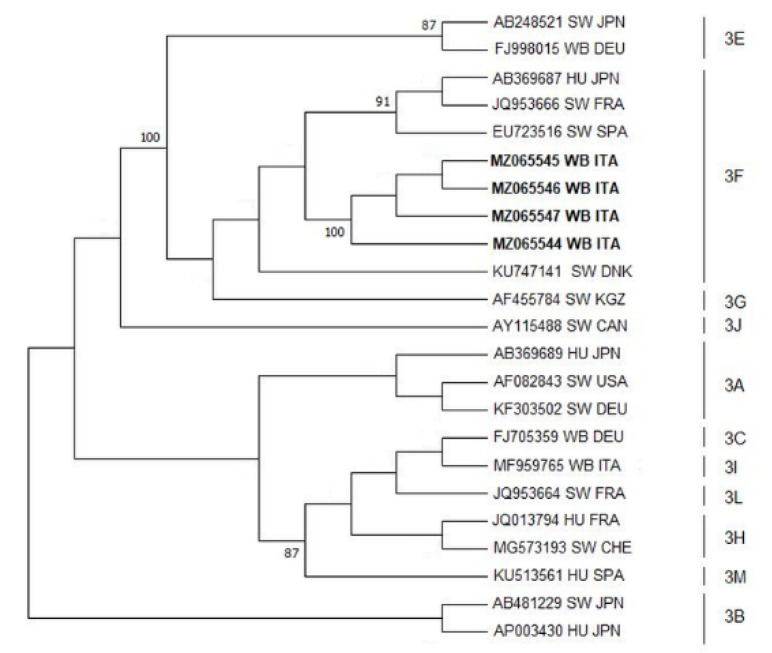
Molecular Phylogenetic analysis by Maximum Likelihood Method: Molecular phylogenetic analysis by Maximum Likelihood method for HEV-3. The evolutionary history was inferred using the Maximum Likelihood method based on the Tamura–Nei model. The percentage of replicate trees in which the associated taxa clustered together in the bootstrap test (100 replicates) is shown next to the branches; bootstrap values lower than 70 have been removed. The analysis involved 23 nucleotide HEV-3 sequences with a total of 347 positions for HEV capsid protein gene in the final dataset. Evolutionary analyses were conducted in MEGA6 [36]. GenBank accession numbers are shown when the available host, host and state are presented. The sequences identified in the present work are represented in bold characters.

The majority of wild boars tested positive for only one of the two sample types, either liver or swab; only two sub-adult males hunted in Roccalbegna (GR) resulted positive for both liver and swab. Positive animals were hunted in 4 different hunting area, 3 in the Province of Grosseto (GR) and 1 in the Province of Prato (PO), and slaughtered on 4 different working days in January 2021 (Table 1). For each positive sample, the viral load expressed as the number of copies of virus on 100 ng of RNA was calculated (Table 1). Mean, standard deviation, maximum and minimum values were calculated for both viral load values of liver samples and viral load values of swabs (Table 1). The standard deviation and the maximum and minimum value show that both within liver samples and swabs, there is a wide variability of values, with values ranging from 11.3 copies/100 ng RNA to 7333 copies/100 ng RNA among livers, and from 38.4 copies/100 ng RNA to 51559 copies/100 ng RNA among swabs. Nested RT-PCR confirmed 9 positive samples, 6 livers and 3 swabs, of which 4 were submitted to sequence analysis for performing phylogenetic analysis. Phylogenetic analysis confirmed the circulation of HEV genotype 3 among the wild boar population of Tuscany. Moreover, the nucleotide sequences obtained from this study were compared to the reference sequences of HEV 3 subtypes (3a–3m), showing higher homology with subtype 3f (Figure 2).

## 4. Discussion

The importance of the wild boar as a potential HEV reservoir and its zoonotic role is a well-known sanitary issue. Indeed, several studies indicate high HEV seroprevalence and virological rates among the wild boar population in Europe. In Italy from 2007 to 2019, a continuous upward trend was recorded of HEV autochthonous cases in humans; in 2019, the number of infections doubled compared to the previous year (102 cases compared to 49 in 2018). Most cases of HEV in humans have been diagnosed in the Abruzzo, Tuscany, Marche and Lazio regions (SEIEVA Bulletin—Epidemiology of Acute Viral Hepatitis in Italy—Update 2021 (EpiCentro)). Moreover, in Italy, and also in Tuscany, the wild boar population is increasing [37] and hunting activity is largely diffused.

In the present study, liver tissue samples and swabs were collected during sanitary inspection and clearance in a slaughterhouse following wild boar hunting. This type of processing is carried out prior to the distribution of wild boar meat among the hunters for local trade and domestic consumption. By real-time assay, we were able to reveal the presence of HEV genome in 12 specimens collected in four different days from 10 wild boar hunted in four different hunting areas, confirming the active circulation of HEV among the wild boar population of Tuscany. Nested PCR identified nine positive samples, likely due to the different HEV genome target identified by the two assays and the higher sensitivity of the fluorescent assay, especially since the samples not identified by nested PCR are those with lower viral loads.

The study found that 12% of liver samples and the 6% of swabs were positive for HEV RNA. The presence of HEV genomes on swabs as well as indications of the contamination of carcass surfaces during slaughtering procedures has proven that the type of swab and the sampling technique was effective. The virological evidence reported in this study is higher than what is described in similar studies previously conducted on domestic swine in different slaughterhouses in Italy. Those studies reported the presence of HEV in pig livers from none to a maximum of 6% [13,15,16,38]. The presence of HEV in livers and contaminated carcasses of wild boar in slaughterhouses could have possible repercussions for both consumers and slaughterhouse personnel health. Interestingly, some swabs resulted positive even though they were collected from animals with negative liver results, indicating probable cross-contamination events among individuals during slaughtering; nonetheless, we cannot exclude the possibility of contamination during pre-processing operations. The viral load analysis of samples indicates a high variability of values on liver and swabs samples with differences in both groups within three orders of magnitude. When considering liver samples, three of them scored a high viral load (from 2079 to 7333 copies/100 ng of RNA), while three samples resulted in a low copy number (from 11 to 53 copies/100 ng of RNA). This variability might suggest that liver scoring low viral load could be cross-contaminated by HEV present in the environment, while liver scoring high viral load could be sampled from infected wild boar. The variability recorded for swabs could be related to the sampling technique, in particular the size of a wild boar carcass and the variability of the degree of contamination of each carcass depending on the source of contamination (handling, utensil, organs, faeces) and the contact with it. Phylogenetic analysis showed high homology among viral amplicons, confirming a unique source of infection. Genotype 3 is confirmed as the genotype circulating among the wild boar population in Tuscany.

## 5. Conclusions

In conclusion, in an area as Tuscany characterised by a diffused wild boar hunting activity, the risk of infection is largely linked to local eating habits, including the traditional consumption of dried liver sausages, and/or in intensive environmental HEV contamination [39]. Furthermore, traditional and homemade food processing is another crucial aspect, and a typical food habit in Mediterranean European countries (Italy, Spain, France, and Greece) [8,9]. However, there is little information concerning HEV survival in food matrices such as ready-to-eat and raw meat products containing wild boar meat and/or liver. Finally, the risk of infection for a person handling infected carcasses and organs cannot be underestimated. Slaughtering procedures, especially those performed on the field, are often carried out without the application of appropriate safety measures for individual protection. Consequently, it is necessary to obtain more data about the viral infectious load from contaminated food consumption and manipulation and to promote health education programmes for hunters and consumers.

## Figures and Tables

**Table 1:** Molecular results of positive wild boar liver and swab.

Animal	Age Category	Sex	Hunting Area	Slaughtering Date	RT-qPCR Viral Load	
					Liver	Swab
1	Adult	F	Semproniano GR	11 February 2021	4.28 × 10^2^	-
2	Adult	F			1.52 × 10^1^	-
3	Adult	M			1.13 × 10^1^	-
4	Adult	M			5.37 × 10^1^	-
5	Adult	F	Orbetello GR	19 February 2021	-	3.84 × 10^1^
6	Adult	F			-	9.9 × 10^2^
7	Sub-adult	M	Roccalbegna GR	25 February 2021	1.31 × 10^3^	1.42 × 10^2^
8	Sub-adult	M			6.12 × 10^2^	5.16 × 10^3^
9	Adult	F			7.33 × 10^3^	-
10	Adult	M	Vicchi PO	31 February 2021	2.08 × 10^3^	-
Mean					1.48 × 10^3^	1.57 × 10^3^
Standard Deviation					2.47 × 10^3^	2.42 × 10^3^

Age, sex, hunting area, slaughtering date and RT–viral load (viral copies/100 ng RNA).
